# Impact of a Student-Led Rheumatology Interest Group on Medical Student Interest in Rheumatology

**DOI:** 10.1155/2019/4892707

**Published:** 2019-02-24

**Authors:** Sonia Silinsky Krupnikova, Timothy Brady, Michael Sheppard, N. Andrew LaCombe, Derek Jones, Victoria K. Shanmugam

**Affiliations:** Division of Rheumatology, Ideas to Health Laboratory, The George Washington University, School of Medicine and Health Sciences, 701 Ross Hall, 2300 Eye Street, NW, Washington, DC 20037, USA

## Abstract

**Objectives:**

This observational study was designed to evaluate the impact of a student-led Rheumatology Interest Group on medical student interest in rheumatology.

**Methods:**

The mean numbers of student-rheumatology interactions per six months were assessed for elective enrollment, abstract submissions, and manuscripts, in the pre- and postinterest group period.

**Results:**

Enrollment in the rheumatology elective increased from 2.0 ± 0.36 per six months in the preintervention period to 6.2 ± 1.24 per six months in the postintervention period (p=0.0064). Abstract submissions increased from 0.5 ± 0.34 to 5.86 ± 1.49 (p=0.0077), and manuscript submissions from 0.16 ± 0.16 to 1.57 ± 0.37 (p=0.074).

**Conclusion:**

The Rheumatology Interest Group significantly increased medical student engagement in rheumatology.

## 1. Introduction

Data from the 2005 and 2016 American College of Rheumatology Workforce Studies shows that, in the coming years, a continued significant increase in demand for rheumatologists is expected [1, 2]; however, the number of trained rheumatologists is declining.

Interest in a certain specialty often begins during medical school. A study completed in the UK in which graduates were surveyed 1 year after medical school found that 45% of graduates reported that “experience of a subject” in medical school influenced their career choice, and 27% reported “exposure to a particular teacher or department” as significantly influencing future career considerations [3]. Many rheumatology fellows report interactions with a mentoring rheumatologist as one of the driving forces behind their decision to select rheumatology as a career [4]. The majority of fellows report initial exposure to rheumatology as a specialty in their second or third year of medical school, and 70% indicate that increased exposure in medical school as well as residency would be the most beneficial means for attracting new rheumatologists into the workforce [4, 5]. Data shows that medical student interest in rheumatology declines from the first and second year compared to the third and fourth year. In light of this, early initiatives to highlight rheumatology as a career, before students have narrowed their focus, might be beneficial in encouraging more students to consider clinical rotations in rheumatology. Senior medical students who had an elective in rheumatology were significantly more likely to report considering it as a possible career option [4, 6].

We sought to investigate the impact of a student-led Rheumatology Interest Group on student uptake of the rheumatology elective, abstract submissions, and manuscript submissions.

## 2. Methods

### 2.1. Interest Group Development

In April, 2015, medical students at George Washington University School of Medicine and Health Sciences (GW SMHS) established a student-led Rheumatology Interest Group. At the inaugural meeting the American College of Rheumatology “Choose Rheumatology” team presented on rheumatology careers, faculty members gave testimonials on why they chose rheumatology, and patients spoke about the impact of their rheumatologist on their lives. Approximately 30 students, 5 faculty members, and 2 fellows attended the first interest group meeting. Follow-up meetings were held 3-4 times per year, with email notification and reminders to all students on the interest group email list to notify them of dates and times. All meetings were held in the early evening at approximately 5 pm in Ross Hall, which is the main School of Medicine and Health Sciences building, and within easy access of the Hospital and Physician Offices. Most meetings consisted of 2-5 faculty members and approximately 15-20 students. Meeting topics included a meeting on identifying mentors and research projects and presentations by patients about the importance of having good clinicians in the field of rheumatology, as well as hands-on joint injection simulation workshops.

### 2.2. Student Leadership of the Interest Group

Students self-selected the interest group leaders each year. This was largely a student-driven process and faculty only assisted in years where no leader was nominated. It should be noted that the George Washington University School of Medicine and Health Sciences does have an Office of Student Professional Enrichment (https://smhs.gwu.edu/oso/student-groups) and student interest groups exist for many of the medical specialties.

### 2.3. Development of Mentor-Mentee Dyads

Students who were interested in linking with a research or career mentor contacted our Rheumatology Project Manager who would then review a brief questionnaire to establish what areas of rheumatology the student might be most suited to. They were then linked with a faculty mentor, and a potential project. Projects varied from data analysis of ongoing research projects, basic science wet-lab research, student initiated research projects, and case report manuscript preparation.

### 2.4. Observational Study

Data was collected from the two years prior to initiation of the student interest group (2012-14) and the three years following initiation of the interest group (2015-18) based on three parameters: rheumatology elective enrollment, medical student abstract submissions to GW Research Day, and manuscripts published. This study did not constitute human subjects research and was thus exempt from IRB approval in accordance with the policy of The George Washington University Office of Human Research. Time of data lock was April 2018.

### 2.5. Statistical Analysis

In order to account for the differing observation periods, data was analyzed using mean number of student-rheumatology encounters per 6 months for each parameter in the pre- and postintervention periods. The mean number of student-rheumatology interactions per six months in the pre- and postintervention periods was assessed for each parameter. Data was analyzed using GraphPad Prism 5.03, with p<0.05 considered significant.

## 3. Results

### 3.1. Rheumatology Elective Enrollment

**Figure 1:**
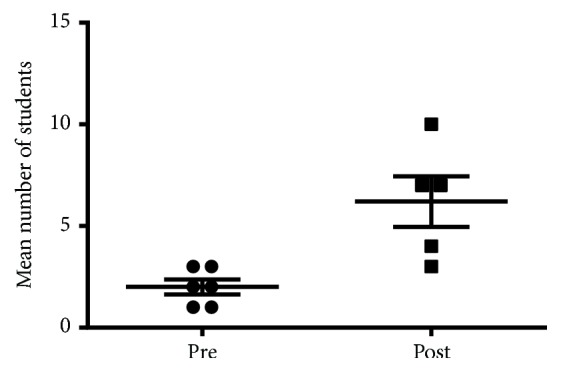
Mean number of students per six months enrolled in rheumatology elective in the time periods prior to and subsequent to development of the Rheumatology Interest Group.

Student enrollment in the rheumatology elective significantly increased following the development of the Rheumatology Interest Group, with a mean number of students per six months of 2.0 ± 0.36 in the preintervention period and 6.2 ± 1.24 in the postintervention period (Figure 1, p=0.0064).

### 3.2. Abstract Submissions

**Figure 2:**
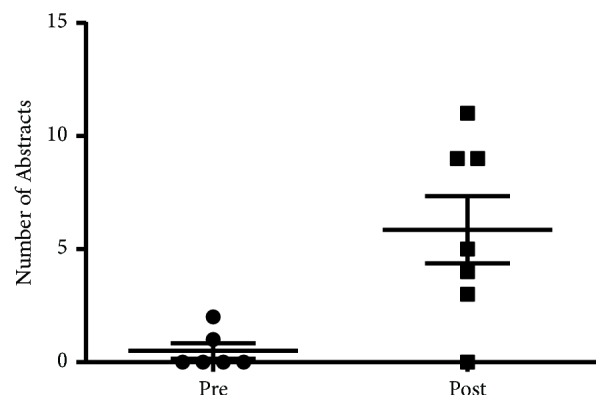
Mean number of abstracts submitted by student-faculty dyads per six months prior to and subsequent to the development of the Rheumatology Interest Group.

The number of abstract submissions also significantly increased with 0.5 ± 0.34 submissions in the preintervention period compared to 5.86 ± 1.49 in the postintervention period (Figure 2, p=0.0077). Abstract topics varied from case reports of interesting cases seen while on the rheumatology elective, data analysis of ongoing projects in the Division of Rheumatology, and, in some cases, wet-lab basic science research. Several students also conducted student initiated research projects that the mentors provided oversight and guidance on as well as access to statistical analysis and other resources as needed. Several students presented abstracts at national and regional meetings.

### 3.3. Manuscripts Submitted by Student-Faculty Dyads

**Figure 3:**
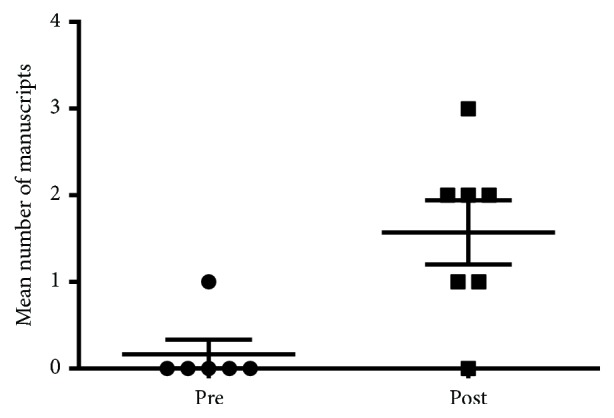
Mean number of manuscripts submitted by student-faculty dyads per six months prior to and subsequent to the development of the Rheumatology Interest Group.

The number of manuscripts submitted per six months by student-faculty dyads has increased since development of the Rheumatology Interest Group from 0.16 ± 0.16 to 1.57 ± 0.37 (Figure 3, p=0.0074). Not all abstracts led to manuscripts but, where possible, students were encouraged to develop their work into a manuscript submission if appropriate. Some students also submitted manuscripts that were never presented as abstracts.

## 4. Discussion

Rheumatology workforce studies confirm that there is an ongoing increasing demand for rheumatologic care, despite lack of growth in the rheumatology workforce [1, 2, 7]. There is a great need to focus efforts on expanding interest in rheumatology as a career among current medical students.

Opportunities to participate in rheumatology electives, clinical rotations, and mentors strongly influence career choice [4, 5, 8]. In this study we sought to investigate the impact of a student-led Rheumatology Interest Group on engagement with rheumatology at a single institution. We focused on several components of the specialty which our faculty found engaging: patient interactions, joint injections and procedures, and immunology research. The interest group intervention provided medical students with increased exposure to rheumatology during preclinical and clinical years as well as access to mentors and research projects. These opportunities generated increased interest and enthusiasm for the field, as evidenced by greater enrollment in the rheumatology elective and increased numbers of abstract and manuscript submissions.

This study has several limitations which merit discussion. Firstly, there are many factors which impact the supply and demand discordance in the rheumatology workforce. While opportunities in medical school are clearly important, interactions during residency, as well as funding and availability of fellowship positions, also impact ultimate career decisions [4, 5, 9, 10]. The true impact of an intervention at the medical student level on the rheumatology workforce could not be assessed in this study and will not be realized for several years. The follow-up time for this interim study was short, but we were able to demonstrate a significant impact of the intervention on student engagement in rheumatology. Longitudinal follow-up of these cohorts over the upcoming years will allow us to track career choices and the impact of this intervention over the longer term.

## 5. Conclusions

In this study we were able to show the short-term impact of the simple and low-cost development of a student-led interest group. We demonstrated increased student engagement as evidenced by increased numbers of publications and increased enrollment in the rheumatology elective. These findings merit further investigation through longitudinal studies. If proven successful, similar interventions would be easily replicated at other institutions and may help to address rheumatology workforce deficits in the years to come.

## Data Availability

Raw data will be made available upon request.

## Additional Points

*Significance and Innovations*. (i) Development of a student-led Rheumatology Interest Group significantly increased student engagement with rheumatology as evidenced by rheumatology elective enrollment, abstract submissions, and manuscript submissions. (ii) This is a cost effective and easily deployed intervention to improve student engagement with rheumatology, which could be harnessed by other institutions within the United States and worldwide to improve student interest in rheumatology.
